# Optimizing implant hygiene: the added value of interproximal cleaning devices around implant-supported restorations—a systematic review and meta-analysis

**DOI:** 10.1186/s40729-025-00652-4

**Published:** 2025-10-20

**Authors:** Shaza Bishti, Stefan Wolfart, Taskin Tuna

**Affiliations:** Department of Prosthodontics and Biomaterials, Centre for Implantology, Uniklinik RWTH Aachen, Pauwelsstrasse 30, Aachen, 52074 Germany

**Keywords:** Dental implant, Restoration, Oral hygiene, Interproximal, Toothbrushing, Peri-implant mucositis

## Abstract

**Statement of problem:**

A well-defined protocol for self-performed interproximal oral hygiene around implant-supported restorations is currently lacking.

**Purpose:**

To assess the efficacy of home-care interproximal cleaning measures used adjunctively to mechanical toothbrushing in reducing signs of inflammation in patients with peri-implant mucositis.

**Methods:**

A systematic electronic literature search followed by a manual search using the PRISMA guidelines was performed. Randomized controlled trials and prospective studies evaluating the effectiveness of different interproximal cleaning devices in reducing inflammation signs in patients with peri-implant mucositis were selected according to pre-determined inclusion and exclusion criteria. The meta-analysis was carried out using the Rev Man 5.4 software program.

**Results:**

Out of a preliminary pool of 792 articles, 6 relevant studies were identified for final evaluation. Interproximal cleaning devices investigated were: dental floss, interproximal brush, oral irrigator(water), oral irrigator(CHX). The following clinical variables were analysed: plaque index(PI), gingival index(GI), bleeding on probing (BOP) and interleukin 6. Due to hetergeneity, only two studies fulfilled the eligibility criteria for meta-analysis. Oral irrigators and interdental brushes showed a higher but not-significant improvement in signs of peri-implant mucositis than dental floss. The meta-analysis revealed no statistically significant difference between toothbrushing alone and toothbrushing with an oral irrigator (water) in reducing BOP and PI.

**Conclusion:**

All interproximal cleaning devices evaluated in this review demonstrated some degree of effectiveness in controlling biofilm accumulation and mitigating peri-implant inflammation. However, evidence on self-performed oral hygiene around dental implants-especially concerning interproximal devices-remains limited.

## Introduction

Innovations in implant dentistry have allowed implant treatment to become a widely accepted therapeutic option for patients with partial or complete edentulism. Studies have reported long-term success and survival rates of more than 95% [1, 2]. However, implant success is not as high as survival, as functioning implants and their restorations may be affected by different technical and biological complications [3]. Biological complications that occur around dental implants are mostly inflammatory conditions of the surrounding soft and hard tissues. These inflammatory conditions have recently been well-defined and distinguished from healthy peri-implant conditions to facilitate the assignment of a proper diagnosis by the clinician and therefore select a proper therapeutic option [4]. As outlined in the 2017 World Workshop on Classification of Periodontal and Peri-implant diseases and Conditions, peri-implant mucositis refers to an inflammatory condition of the soft tissues surrounding an endosseous implant in the absence of loss of supporting bone, mainly characterized by bleeding on probing as well as visual signs of inflammation [5, 6]. On the other hand, peri-implantitis was defined as a plaque‐associated pathological condition affecting the tissues around dental implants, characterized by inflammation in the peri‐implant mucosa and subsequent progressive loss of supporting bone [5, 7]. Peri-implant mucositis is reversible, however, in the absence of treatment, it can progress to peri-implantitis [5, 7, 8]. Causative factors of peri-implant disease include bacterial flora, biomechanical factors such as excessive occlusal forces, systemic health conditions like diabetes mellitus, lifestyle-related aspects including poor oral hygiene and smoking, parafunctional habits such as bruxism, and iatrogenic factors such as lacking primary stability and premature loading during the healing period [9, 10]. Plaque accumulation on dental implant surfaces is regarded as a main etiological factor in the development of peri-implant mucositis and peri-implantitis [8, 11]. Clinical trials have reported greater prevalence of peri-implant mucositis especially in patients with poor overall oral hygiene [12]. In addition, the patient’s capability and/or accessibility to perform proper implant hygiene highly influences the likelihood of developing peri-implant diseases [13].

In fact, evidence has shown that brushing alone may eliminate only around 60% of overall plaque per routine cleaning session [14]. Slot et al. estimated that the efficacy of plaque removal around teeth following a brushing episode averages around 42% [15]. Brushing is also thought to be more optimal for cleaning flat surfaces such as buccal/lingual surfaces of teeth compared to interproximal surfaces, which highlights that toothbrushing alone is insufficient and must be complemented by interproximal tools [16]. A variety of dental implant hygiene devices are available on the market, yet clear guidelines for effective homecare around implants remain scarce. Previous reviews have addressed nonsurgical management of peri-implant mucositis and peri-implant diseases [17–20]. Barootchi et al. performed a systematic review and meta-analysis of nonsurgical interventions, highlighting that while both professional and patient-administered therapies can reduce inflammation, the heterogeneity of study designs and outcomes limits firm conclusions [17]. Similarly, Heitz-Mayfield et al. provided a comprehensive narrative review of peri-implant mucositis management, discussing both professional mechanical debridement and patient oral hygiene, and highlighting that variability in study protocols, prosthetic design, and treatment settings limits the comparability of results and underscores the need for more standardized clinical trials [6]. More recently, Ghandi et al. concentrated on oral irrigators compared with other interdental aids, reporting potential benefits but restricting inclusion to this single device category and also considering removable overdentures [21]. Collectively, these reviews illustrate the accumulated evidence on peri-implant hygiene approaches, but none directly evaluated randomized or prospective controlled trials of self-administered interproximal aids compared with toothbrushing alone.

Therefore, this systematic review aims to summarize the different interproximal cleaning devices for dental implants and to assess how effectively these devices reduce plaque and inflammatory signs in patients with peri-implant mucositis.

## Materials and methods

This systematic review was designed according to the Preferred Reporting Items for Systematic Review and Meta-analysis (PRISMA) statement [22, 23] and the Cochrane Handbook for Systematic reviews (version 6.2, 2021).

### Eligibility criteria

The eligibility criteria for participants, interventions, comparisons, and outcomes were defined as follows:

- **(P)** Population: Patients with implant-supported restorations showing signs of peri-implant mucositis.
- **(I)** Intervention: The use of homecare interproximal hygiene devices around the implant restorations.
- **(C)** Comparison: The use of manual or electric toothbrush alone.
- **(O)** Outcome: Reduction in signs of inflammation, plaque index, probing depth, interleukin levels in patients with peri-implant mucositis.

In alignment with the above- mentioned PICO criteria, the following inclusion and exclusion criteria were applied:

## Inclusion criteria

- Human clinical studies including randomized clinical trials and prospective studies.
- Clinical studies with a minimal sample size of 10 patients.
- Clinical studies including patients with single- or multi-unit implant-supported restorations showing signs of peri-implant mucositis.
- Clinical studies reporting on the efficacy of using interproximal hygiene devices by measuring the plaque index, bleeding on probing (BOP), probing index, interleukin levels.

## Exclusion criteria

- Retrospective studies, case reports, animal studies and in-vitro studies.
- Clinical studies including patients with removable implant-supported restorations.
- Clinical studies including patients with peri-implantitis.
- Studies where the reported data of teeth and implants are pooled together.

## Search strategy

Based on the PICO criteria, a search strategy was developed and executed using an electronic search. An electronic search of PubMed MEDLINE, Cochrane Central Register of Controlled Trials (CENTRAL), including the gray literature of Google Scholar was carried out for clinical studies up to February 2024. No restrictions were placed on language or publication date. An additional manual search was performed identifying relevant studies by screening the reference list of all obtained full-text articles.

## Search protocol

The search pattern was categorized based on the PICO framework into population, intervention, comparison, and outcome. For each category, a combination of Medical Subject Headings (MeSH Terms) and free-text words was used in simple or multiple conjunctions (Table 1).

**Table 1:** List of terms (MeSH and free text) used for the electronic research

Category	Keyword	MeSH terms	Text word
Population	Implant	Dental Implant OR dental implants	Implant OR implants AND dental
	Restoration	Dental Prosthesis OR Dental prosthesis, crown, dentures	Implant-supported, superstructure OR fixed AND reconstruction OR restoration
Intervention	Oral hygiene aids	Oral hygiene	Interproximal AND hygiene AND selfcare OR homecare OR self-administered AND device OR aid OR instrument AND interdental brush OR dental floss OR water flosser OR water pik OR oral irrigation OR mouthwash OR air flosser OR superfloss
Comparison	Toothbrushing	Toothbrushing	Toothbrush AND manual AND electric
Outcome	Peri-implant mucositis treatment	—	Peri-implant mucositis AND peri-implant crevicular fluid AND plaque index AND bleeding on probing OR BOP AND peri-implant biomarkers AND probing depth AND inflammation AND efficacy

### Selection of studies

Two reviewers (SB and TT) independently screened all retrieved titles and abstracts against the predefined outcomes. Any discrepancies were resolved through discussion. Abstracts meeting the initial inclusion criteria were re-screened for confirmation, and full-text articles were obtained for all eligible studies. When titles or abstracts lacked sufficient detail, full texts were also retrieved. Final inclusion was determined according to the specified inclusion and exclusion criteria, with all disagreements resolved by consensus.

## Data extraction and method of analysis

A standardized data extraction form was used to collect and tabulate data for subsequent analysis, with any discrepancies resolved through discussion. The collected information included the author(s), publication date, and study design, as well as sample size in terms of the number of patients and implants. Details on the implant type, implant system, and jaw localization were recorded, along with the type of restoration, retention method, and restoration material. The control group was categorized based on the type of toothbrush used, while the test group was classified according to the interproximal cleaning device or brand. Outcome measures included plaque index (PI), probing depth (PD), bleeding on probing (BOP), and interleukin levels.

The reported results of the studies were categorized by the type of interproximal cleaning device and the predefined patient-level outcomes, and when possible, a meta-analysis was performed. Missing information from the included studies was requested via email from the corresponding authors. Studies were excluded if the required data were not provided.

## Quality assessment

Risk of bias in individual studies was assessed using the Cochrane Collaboration tool for randomized controlled trials (RCTs), evaluating random sequence generation, allocation concealment, blinding, completeness of outcome data, and selective reporting. For non-randomized studies, the ROBINS-I tool was applied, assessing bias related to confounding, participant selection, intervention classification, outcome measurement, and selection of reported results. According to the ROBINS-I criteria, the overall risk of bias for included studies was categorized as “Low,” “Moderate,” or “High”.

### Statistical analysis

A meta-analysis was conducted using random-effects models to compare mean outcome values between the control group (toothbrushing alone) and the test group (toothbrushing combined with an interproximal cleaning aid). Interstudy heterogeneity was evaluated using the DerSimonian–Laird estimate (τ²) for between-study variance and the I² statistic, with 95% confidence intervals. Results were displayed as forest plots. All statistical analyses were performed using Review Manager (RevMan) version 5.4 (The Cochrane Collaboration, 2020).

### Strength of evidence

The certainty of evidence for each outcome was evaluated using the Grading of Recommendations, Assessment, Development and Evaluation (GRADE) framework. Under this system, randomized controlled trials (RCTs) begin as “high certainty” evidence but may be downgraded by one or more levels—to “moderate,” “low,” or “very low” certainty—based on five potential limitations: (i) risk of bias, (ii) indirectness, (iii) inconsistency, (iv) imprecision, and (v) publication bias. The initial GRADE assessments were conducted by one reviewer (SB) and independently verified by a second reviewer (TT).

## Results

### Study selection

**Fig. 1:**
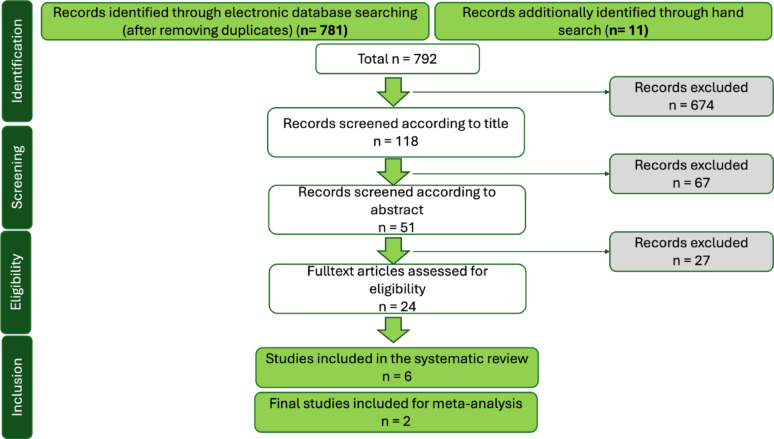
Flowcart of the systematic search results

The electronic search yielded 781 records, with an additional 11 studies identified through manual searching. Following title screening, 674 records were excluded. The remaining 118 studies underwent abstract screening, resulting in 24 being selected for full-text review. After applying the inclusion and exclusion criteria, 6 studies were deemed eligible for data extraction [24–29]. Considerable heterogeneity in research designs and methodologies was observed among these studies; consequently, only two met the criteria for inclusion in the meta-analysis. The study selection process is summarized in Fig. 1, while Table 2 details the excluded studies along with the reasons for their exclusion.

**Table 2:** Final excluded studies and their reason for exclusion

Study	Reason for exclusion
Chongcharoen et al. [30]	Interdental oral hygiene was performed by a dental assistant and not the patient
Luz et al. [31]	No differentation between teeth and implants.
Maeda et al. [32]	The cleaning surface of removable prostheses were investigated extraorally.
Pons et al. [33]	Clinical parameters were evaluated according to the accessibility and not according to the type of interproximal cleaning aid used.
Setti et al. [34]	Pilot study investigating an angled implant brush not considered as an interproximal cleaning aid.

The included studies were published between 2013 and 2024, and were all randomized controlled trials. The interproximal cleaning aids investigated in the included studies were: dental floss (4 studies) [24, 25, 27, 28] interproximal brush /4 studies) [24, 25, 28, 29], oral irrigator with water (4 studies) [24, 26, 27, 29] and chlorhexidine (CHX) (1 study) [26]. The clinical parameters evaluated included plaque index, gingival index, bleeding on probing and interleukin-6.

Due to the lack of a control group in four of the included studies, only a descriptive analysis was possible [24, 25, 27, 28].

### Study characteristics

Table 3 provides a summary of the included studies, all of which were parallel-design RCTs. In two of these trials, the follow-up period was limited to a maximum of 14 days [24, 25], 30 days in one study [27], 3 months in two studies [26, 29] and 6 months in one study [28].

**Table 3:** Mean characteristics of the final included studies

Study name	Study design / Follow-up period	Sample size (patient /implant)	Jaw location	Type of implant/ restoration	Control group	Type of toothbrush	Interproximal cleaning aid	Brand	Outcome (Baseline/Follow-up)			
									PI	BOP	GI	IL-6 (ng/L)
Almoharib et al. [24]	RCT / 14 days	45/45	Mandibular posterior	NA / Single crown	NA	Manual	Interproximal brush	Oral B Proxy brush, Procter & Gamble, USA)	NA	NA	20% Type II /100% Type I	58.38 ± 3.24 / 60.74 ± 2.93
							Water flosser	H_2_O Floss, Shenzen BFT Electrical appliances, China	NA	NA	6.6% Type II /100% Type I	60.17 ± 3.07 / 58.79 ± 4.02
							Dental floss	Oral B Glide Floss	NA	NA	6.6% Type II /100% Type I	54.60 ± 12.22 / 55.70 ± 10.65
Bevilacqua et al. [25]	RCT / 2 weeks	38/38	Maxillary posterior	Titanium, sand-blasted and acid-etched / Single crown	NA	24 Manual / 14 Electric	Interproximal brush	DF TePe Interdental Brush Original, TePe Mundhygienprodukter AB, Malmö, Sweden	M: 1.73 ± 0.83 / 0.95 ± 0.79 D: 1.82 ± 0.85 / 1.09 ± 0.68	NA	M: 1.77 ± 0.69 / 0.64 ± 0.78 D: 1.95 ± 0.65 / 0.95 ± 0.58	NA
							Dental floss	TePe Bridge & Implant Floss (TePe Munhygienprodukter AB, Malmö, Sweden)	M: 1.73 ± 0.83 / 0.91 ± 0.75 D: 1.82 ± 0.85 / 0.91 ± 0.69	NA	M: 1,77 ± 0.69 / 0.64 ± 0.66 D: 1.82 ± 0.85 / 0.86 ± 0.64	NA
Bunk et al. [26]	RCT / 4,8 and 12 weeks	60/60 (the implant with the highest severity of peri-implant mucositis was investigated	NA	NA / Single crown	Toothbrushing alone without any further oral hygiene aids	Manual	Toothbrush alone (No interproximal cleaning aid	(Curadent Germany GmbH, Curaprox CS 5460	1.33 ± 0.52 / 1.06 ± 0.43	2.35 ± 0.99 / 1.20 ± 0.77	NA	NA
							Oral irrigator with water	(Waterpik^®^ Cordless Plus Water Flosser WP-450, Waterpik^®^ Plaque Seeker^®^ Tip, Waterpik Inc.) + (B. Braun Melsungen AG, B. Braun aqua)	1.19 ± 0.49 / 1.00 ± 0.67	2.25 ± 1.02 /1.15 ± 1.18		
							Oral irrigator with 0.06% CHX solution	(Waterpik^®^ Cordless Plus Water Flosser WP-450, Waterpik^®^ Plaque Seeker^®^ Tip, Waterpik Inc.) + (B. Braun Melsungen AG, B. Braun aqua)	1.26 ± 0.40 / 1.03 ± 0.60	2.40 ± 0.88 / 0.75 ± 0.97		
Magnuson et al. [27]	RCT / 30 days	28 / 40	NA	NA / NA	Toothbrushing alone without any further oral hygiene aids	Manual	Toothbrush alone (No interproximal cleaning aid)	Oral-B^®^ Soft Compact 35, Procter & Gamble	NA	NA	NA	NA
							Dental floss	(Reach^®^, Johnson & Johnson Oral Care Company)		33.3% reduction in BOP		
							Water flosser	(Waterpik^®^ Ultra Water Flosser, Water Pik, Inc.)		81.8% reduction in BOP		
Nwachukwu [28]	RCT / 3 and 6 months	32 /32	NA	NA / Single crown	NA	Manual (TePe manual toothbrush, TePe Munhygienprodukter AB, Sweden)	Dental floss	TePe Bridge & Implant Floss (TePe Munhygienprodukter AB, Malmö, Sweden)	NA	Not clearly defined	Not clearly defined	166.6 /21.16 (6 m)
							Interproximal brush	DF TePe Interdental Brush Original, TePe Munhygienprodukter AB, Malmö, Sweden				40.38 / 81.88
Tütüncüoğlu et al. [29]	RCT / 2, 4 and 12 weeks	45/45	NA	NA/NA	Toothbrush alone with no interproximal cleaning aid	Manual	Toothbrush alone with no interproximal cleaning aid	Oral-B^®^ Soft Compact 35, Procter & Gamble, Gross-Gerau, Germany	1.67 ± 0.49 / 0.67 ± 0.49 (12w)	0.93 ± 0.26 / 0.47 ± 0.52	NA	NA
							Interdental brush	Oral-B^®^ Pro-Expert Clinic Line Interdental starter kit, Germany	1.80 ± 0.41 / 0.67 ± 0.26	1.00 ± 0.00 / 0.00 ± 0.00	NA	NA
							Oral flosser	Oral-B^®^ Professional Care MD20 Oxyjet Oral Irrigator, Germany	1.73 ± 0.46 / 0.33 ± 0.49	1.00 ± 0.00 / 0.00 ± 0.00	NA	NA

RCT: Randomized controlled trial, NA: Not applicable, PI: Plaque index, BOP: Bleeding on probing, GI: Gingival index, IL6: Interleukin 6, M: mesial, D: distal, CHX: chlorhexidine

Three out of the six studies presented 3 arms [24, 26, 29]. The remaining studies compared the efficacy of two interproximal cleaning aids with no control group.

The test groups were categorized based on the type of interproximal cleaning device, yielding a total of four comparisons. Among these, the most frequently evaluated interproximal hygiene aids were the water flosser (*n* = 4), interproximal brush (*n* = 4) and dental floss (*n* = 4). In one study, the oral flosser with CHX was considered [26].

### Participant characteristics

#### General characteristics

Overall, six studies with a minimum follow-up of 14 days included a total of 248 participants, with group sizes ranging from 15 to 20 individuals. Participant ages spanned from 23 to 89 years. Three of the studies included both smokers and non-smokers [25, 28, 29]. , while smoking was an exclusion criterion in three studies [24, 26, 27]. In one study, only heavy smokers—defined as individuals consuming more than 10 cigarettes per day—were excluded [25].

The periodontal diagnosis of the included participants was inadequately reported. One study excluded patients receiving periodontal treatment within the last month prior study start [24]. In two studies, only individuals participating in routine maintenance care programs were included and were thus assumed to be periodontally healthy [26, 28]. The other three studies, however, did not report any details regarding the periodontal condition of their subjects [25, 27, 29].

#### Definition of peri-implant mucositis

The definition of peri-implant mucositis varied across the included studies. Only one study [29] adhered to the criteria outlined in the consensus report of the VII European Workshop on Periodontology [6]. In two studies, peri-implant mucositis was primarily defined by probing pocket depths of up to 5 mm in the absence of radiographic bone loss [24, 28], whereas bleeding on probing was the characteristic of definition in three other studies [25–27].

#### Number, type and position of dental implants

All included studies reported clinical data for a single implant per patient. Two studies further restricted inclusion to implants that had been in function for a minimum of 24 months [29], and 4 years [24]. The remaining four studies provided no information about how long the implants have been placed prior to study participation. With the exception of one study [25], which reported titanium implants with sandblasted and acid-etched surfaces, the implant brands were not specified in the included studies. Implant dimensions, including width and length, were also generally unreported. Concerning implant location, one study indicated that the implants were placed in the mandible [24], one study in the maxillary posterior region [25], whereas the rest of the studies provided no information related to implant position.

#### Type of restorations

Only five trials reported on the type of restoration in which four studies investigated single crowns [24–26, 28]. Out of the six included trials, only one study included both cemented and screwed restorations [25]. All other studies provided no information regarding type of restoration retention.

### Type of interdental cleaning device

#### Dental Floss

In four studies, the use of dental floss was examined for its ability to manage plaque accumulation and inflammation around implants [24, 25, 27, 28]. Owing to the heterogeneity of the investigated parameters and the absence of control groups in all studies, a meta-analysis could not be performed.

Almoharib et al. [24] compared the IL-6 parameter levels at baseline and after two weeks of using dental floss. A non-significant elevation in IL-6 levels was observed in the second visit (55.7 ± 10.64 ng/L) compared to the mean level recorded at the first visit (*p* = 0.760). During the first visit, 20% of the participants had a type II ginigival index. The use of dental floss for 14 days reported decrease in bleeding on probing and improvement in gingival health resulting in 100% of patients having a type I gingival index after two weeks.

Comparable findings were reported by Nwachukwu et al. [28], who observed a non-significant trend toward reduced IL-6 levels following the use of dental floss over 3 and 6 months.

In the study by Bevilacqua et al. [25], the impact of dental floss on biofilm removal and management of peri-implant mucositis was investigated. The plaque index around the analyzed implants after 2 weeks of dental floss use was 0.91 ± 0.69 for the distal surface and 0.91 ± 0.75 for the mesial surface and was statistically significant (*p* < 0.01). The gingival index (GI) was 1.95 ± 0.65 on the distal surface and 1.77 ± 0.69 on the mesial surface, respectively. After 2 weeks, GI lowered to 0.86 ± 0.64 for the distal surface (*p* < 0.0001) and to 0.64 ± 0.66 for the mesial surface (*p* < 0.0001). Here, a statistically significant relationship between the manual dexterity of dominant hand of the patient and GI was reported when a dental floss was used.

**Fig. 2:**
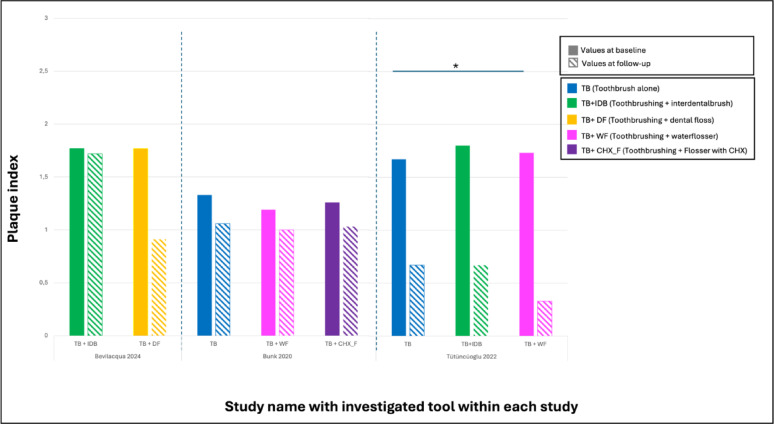
Chart demonstrating the plaque index investigated in the studies with the tools investigated in each study. Only one study (Tütüncüoglü 2022) reported a statistically significant difference between toothbrush alone and toothbrush with water flosser

**Fig. 3:**
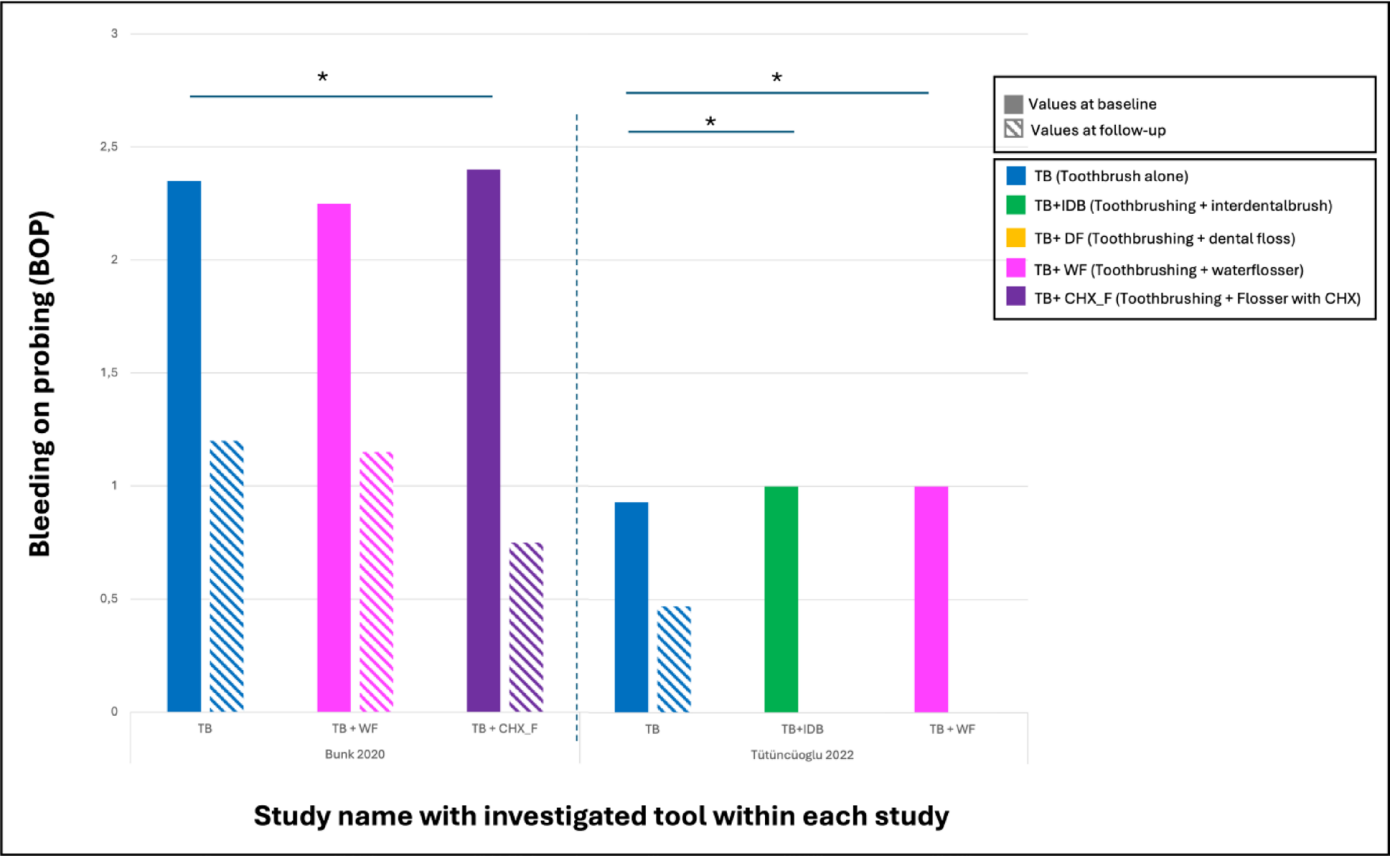
Chart demonstrating bleeding on probing (BOP) investigated in the studies with the tools investigated in each study. Statistically significant differences are marked with *. Note that both groups in (Tütüncüoglü 2022) showed 0 values at the follow-up period

Magnuson et al. [27] measured the reduction in BOP after 30 days of using dental floss. Here, only 33.3% experienced a reduction in BOP after 30 days, which was considered statistically non-significant (Figs. 2 and 3).

### Interdental brush

Four studies evaluated the effectiveness of interdental brushes in managing peri-implant mucositis [24, 25, 28, 29]. However, due to the absence of control groups in three of the studies and heterogeneity in the investigated parameters, no meta-analysis was possible.

One study reported a non-significant rise in IL-6 levels after two weeks of interproximal cleaning with an interdental brush [24]. Similar results were reported after 3 and 6 months. Again, no-significant differences could be seen [28]. In the first study, 40% of the participants had type II gingival index during the first visit. Enhancements in bleeding on probing after two weeks of using an interdental brush was seen, this resulted in 100% of participants having a type I gingival index at the end of the investigation [24].

Another study reported significant decrease in plaque and gingival levels on the interproximal surfaces of implants after 14 days of interproximal cleaning using an interdental brush [25]. Here, other factors such as manual dexterity and interproximal emergence angle were investigated for their relationship with the amount of plaque accumulation. Both factors seemed to have no influence when the interproximal brush was used.

Among the included studies, only one directly compared the efficacy of an interdental brush to a control group, in which participants used solely a manual toothbrush without any supplementary interproximal cleaning device [29]. The interdental brush reduced levels of modified plaque index, bleeding on probing and probing pocket depth after 12 weeks of use. However, these changes were statistically non-significant (Figs. 2 and 3).

### Oral irrigator

The impact of oral irrigators on reducing peri-implant mucositis was investigated in four studies [24, 26, 27, 29]. All studies investigated oral irrigators with water (water flosser), whereas only one study evaluated an oral irrigator with CHX instead of water [26]. As only two studies included a control group (with only a toothbrush and no interproximal cleaning device), a meta-analysis with these two studies was possible [26, 29].

Only one study measured the proinflammatory cytokine level (IL-6) before and 14 days after use of a water floss. Here, a non-significant decrease in the mean IL-6 level at the second compared to the first visit (60.17 ± 3.07 vs. 58.8 ± 4.04, respectively) was reported. In another study measuring the incidence of bleeding on probing after 30 days of water flosser use, 81.8% of the implants experienced a reduction in BOP. This 2.45-fold difference (145%) was highly statistically significant (*p* = 0.0018) [27].

When comparing oral irrigator with water and oral irrigator with 0.06% CHX, patients showed significantly lower BOP-positive sites after 12 weeks when compared to the control group (*p* = 0.004). No significantly lower BOP-positive sites could be found when oral irrigation with CHX was compared to water (*p* = 0.16). Moreover, a significant improvement of the severity of peri-implant mucositis when using CHX irrigation compared to toothbrushing alone (*p* = 0.001) was reported. The use of an irrigation device with water compared to control resulted in an estimated drop of 1.7 points in mucositis severity score after 12 weeks. This was statistically not significant (*p* = 0.06) (Figs. 2 and 3).

### Synthesis of the results from meta-analyses: 12-month data

Due to study heterogeneity and incomplete data, only two studies were eligible for meta-analysis. For the statistical analysis, only outcomes reporting mean values with standard deviations were included.

#### Modified plaque index (PI)

**Fig. 4:**
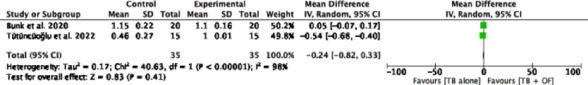
Forest plots showing the mean difference in modified plaque index between control (toothbrushing alone) and test (toothbrushing + water flosser) using the random effect model, no significant difference could be detected

Pooled analysis using a random-effects model indicated that combining toothbrushing with a water flosser did not result in a statistically significant difference compared to toothbrushing alone. A total mean difference of 0.2 could be detected between the control and test group, in favor of the control group (toothbrushing alone). The meta-analysis showed 68% level of heterogeneity between the studies. Figure 4 shows a forest plot with the results of mean differences in modified plaque index between toothbrushing alone and toothbrushing with waterflosser.

#### Bleeding on probing (BOP)

**Fig. 5:**
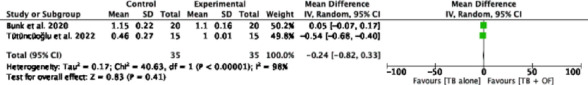
Forest plots showing the mean difference in bleeding on probing between control (toothbrushing alone) and test (toothbrushing + water flosser) using the random effect model, no significant difference could be detected

The pooled analysis of the random effect model showed no statistically significant difference between toothbrushing alone and toothbrushing combined with waterflosser. A total mean difference of 0.24 could be detected between the control and test group, in favor of the control group (toothbrushing alone). The meta-analysis revealed 98% level of heterogeneity between the studies. Figure 5 shows a forest plot with the results of mean differences in bleeding on probing between toothbrushing alone and toothbrushing with waterflosser.

### Risk of bias in the included studies

**Fig. 6:**
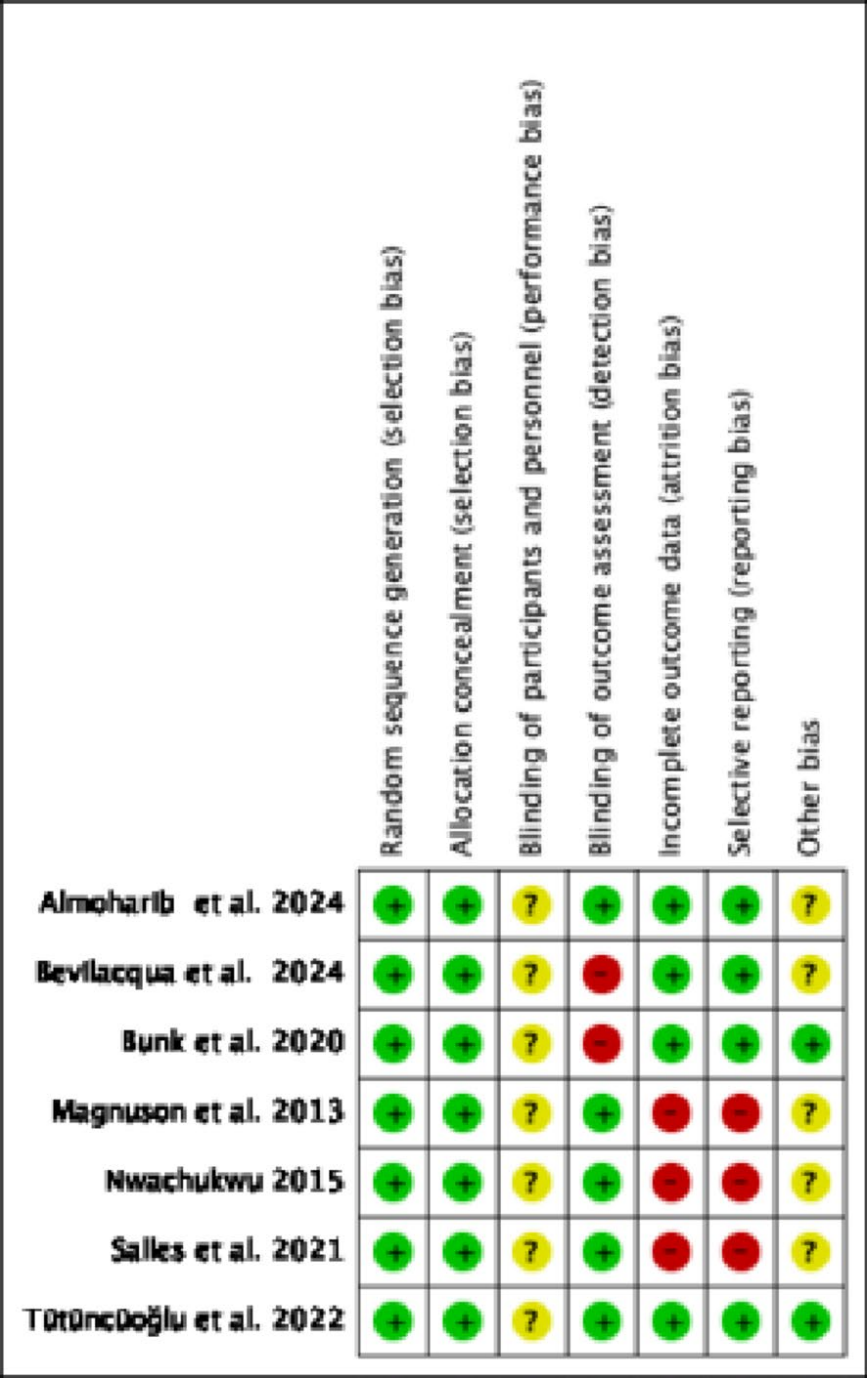
Assessment of risk of bias in the randomized clinical trials using Cochrane Collaboration tool

Figure 6 presents a summary of the risk of bias assessment for the six studies included in this review. The methodological quality of the randomized clinical trials was evaluated using the Cochrane Collaboration tool. Each study exhibited at least one domain with a high or unclear risk of bias. Performance bias was rated as unclear in all the included studies as blinding of participants was not possible since the interproximal cleaning aids were used during their home care oral hygiene protocol. At least three studies demonstrated a high risk of attrition bias due to incomplete outcome data reporting. In contrast, all included studies were assessed as having a low risk of selection bias.

### Strength of evidence

Across both overall and subgroup analyses, no evidence of publication bias was observed for any of the outcomes. Downgrading of quality ratings was mainly attributed to substantial unexplained heterogeneity (i.e., inconsistency of results) and the short duration of follow-up. For studies with follow-up periods up to three months, the overall strength of evidence was considered low, whereas studies exceeding six months demonstrated moderate to high evidence quality.

The primary outcome of the modified plaque index was rated as ‘moderate quality,’ while bleeding on probing (BOP) was assessed as ‘low quality,’ reflecting the high heterogeneity (98%) and inconsistency across included studies. When evaluating each adjunctive intervention separately, the evidence was predominantly rated as ‘low’ for shorter-term studies (0–3 months).

## Discussion

This systematic review aimed to evaluate the effectiveness of self-administered interproximal oral hygiene measures, used alongside mechanical toothbrushing, compared to toothbrushing alone, in reducing clinical signs of peri-implant mucositis. The findings revealed that peri-implant mucositis was frequently either undefined or reported inconsistently across studies. None of the included studies documented complete resolution of peri-implant mucositis through self-administered oral hygiene measures. Consequently, the effectiveness of self-performed interproximal hygiene aids—including dental floss, interdental brushes, and oral irrigators—remains inconclusive.

The study strategy of the current systematic review aimed to narrow the inclusion criteria as much as possible to increase homogeneity among the included studies. However, despite the strict search strategy adopted in this review, heterogeneity was one of the main reasons which hampered the conduction of a meta-analysis using all the included studies. Moreover, this review confirmed the huge lack of well-structured clinical trials investigating the efficacy of interproximal hygiene aids around implant restorations, making it impossible to reach a consensus regarding the most effective cleaning device for plaque removal in interproximal areas.

Water flosser was the most common interproximal cleaning device evaluated in the literature, followed by dental floss and interdental brushes. All included studies comparing the effectiveness of water flossers with dental floss or interdental brushes indicated that water flossers may be superior to dental floss or interdental brushes in decreasing clinical indicators of peri-implant mucositis, such as inflammation and plaque levels. These results are in line with a Cochrane review published in 2019, which reported superiority of water flossers over interdental brushes and dental floss in reducing bleeding on probing [35]. According to the meta-analysis conducted in the current review, no statistically significant difference could be seen when comparing water flosser to mechanical toothbrushing alone, in reducing plaque index levels and bleeding on probing. However, caution is warranted in interpreting these findings, given that only two studies were incorporated into the meta-analysis and the observed heterogeneity was substantial (98%).

Regarding the effectiveness of interdental brushes and dental floss, the descriptive analysis in this review revealed no significant differences between these devices in interproximal plaque removal. These findings align with a randomized clinical trial that evaluated the impact of interdental brushes and dental floss on bleeding on probing over a 12-week period, which reported no difference in plaque reduction between the two methods [36]. Similarly, a study involving ten patients with periodontal disease demonstrated comparable improvements in plaque scores for both devices [37]. Furthermore, a previous systematic review concluded that most available studies do not provide robust evidence supporting the superiority of flossing over other interdental plaque control methods [38].

An additional consideration with interdental brushes is the importance of selecting the correct size for each interproximal space. While it is generally recommended to use the largest brush diameter that can comfortably fit without causing trauma [39], none of the randomized controlled trials included in this review stratified outcomes according to brush size. This represents a limitation in the available evidence, as brush diameter may influence both efficacy and patient acceptance.

Although dental floss remains the most frequently used tool for interdental hygiene, it is not without drawbacks. Three case reports in the literature have described floss as a potential contributing factor to periodontal and peri-implant inflammation [40–42]. Retained floss remnants in the peri-implant sulcus have been implicated in localized inflammation and subsequent marginal bone resorption [41, 42]. It was assumed that initial bone loss gradually exposes rough implant surfaces, allowing dental floss fibers to become trapped in the peri-implant sulcus, which in turn may trigger peri-implant infection [42]. Discontinuation of flossing in the oral hygiene regimen led to resolution of the condition [40]. Therefore, the use of dental floss is not recommended in regions where rough implant surfaces are exposed or in situations prone to shredding. In such cases, interdental brushes or oral irrigators may serve as safer and more effective alternatives for interproximal cleaning.

Among the included studies, only one assessed the effect of chlorhexidine-based oral irrigation on peri-implant mucositis, with a follow-up duration of 12 weeks [26]. Irrigation with 0.06% CHX showed a significantly higher reduction of peri-implant mucositis compared to patients with no irrigation or those with water irrigation [26]. Recent studies have been concentrating on antibacterial substances for reducing the inflammatory process of peri-implant tissues [43]. Despite its side effects, such as staining of the intraoral tissues, hypersensitivity, xerostomia, mucosal irritations, or allergic reactions, CHX is increasingly being used in the clinical practice due to its antimicrobial effect. Daily rinsing with a 0.03% chlorhexidine solution resulted in a more pronounced reduction in bleeding on probing at buccal implant sites compared to other mouth rinses, suggesting superior antimicrobial and detoxifying efficacy relative to agents such as cetylpyridinium chloride, hydrogen peroxide, and citric acid [44–46]. However, the efficacy of CHX as a mouthwash differs from its use with an oral irrigator in reducing signs of peri-implant mucositis. In a study comparing an oral irrigator containing 0.06% chlorhexidine with a 0.12% chlorhexidine rinse, the use of the oral irrigator resulted in a significant decrease in both the modified gingival index and bleeding index [43].

In general, other factors such as accessibility, manual dexterity, restoration design and time seem to play a much more important role than the interproximal cleaning device itself. Adequate access to peri-implant areas is essential, since compromised interproximal hygiene has been correlated with peri-implant disease, especially mucositis. Studies indicate that insufficient access negatively affects peri-implant soft tissues, leading to greater mucosal inflammation and elevated full-mouth bleeding indices [33]. A clinical retrospective study has shown that a significant number of implants that had developed peri-implantitis were associated with a lack of access for appropriate plaque control [13]. Moreover, it has been observed that patients with limited access for interproximal hygiene and consequently a higher prevalence of peri-implant disease, are generally aware of their condition. Greater discomfort, pain, and difficulties while performing oral hygiene was reported by these patients. In addition, patient compliance with guidance on peri-implant oral hygiene was largely inadequate. Furthermore, pre-treatment education concerning peri-implant diseases and their risk determinants was frequently missing [33]. A clinical study examining the relationship between patients’ access to self-performed interproximal hygiene and peri-implant health found that individuals with limited access to hygiene measures exhibited a markedly higher prevalence of peri-implant diseases compared to those with adequate access [33]. These results align with previous research showing that nearly half of implants with peri-implantitis were in patients unable to maintain adequate oral hygiene, whereas only 4% occurred in patients with good hygiene access [13]. Similarly, 77.2% of patients with peri-implantitis reported limited or no access for oral hygiene [47]. Interestingly, adjustments to prosthetic contours were strongly linked to the improvement of peri-implant mucositis [48].

Manual dexterity and additional time required for interproximal cleaning are also factors known to influence mechanical plaque control, especially in the interproximal areas [37, 49]. Up to now, there is no standard of care for the interdental cleaning of implants and the search for alternative methods that are easier for the patient would increase patient comfort and motivation. Oral irrigators are believed to be the more controlled and easier-to-use device when compared to other interproximal cleaning aids. In a randomized clinical trial evaluating the success of oral irrigators in the management of peri-implant mucositis after an follow-up time of 12 weeks, superior clinical and biochemical parameters were reported, when compared to interdental brushes [29]. In another study assessing the effectiveness of two different oral irrigators, 57.4% of the patients were observed to have continued using the oral irrigator till the end of the 1-year follow-up period of the study [50]. In another 6-month investigation, patient compliance with oral irrigator use reached 90.6% [51]. In agreement with the above-mentioned studies, the successful results achieved with the oral irrigators may be attributed to the fact that the device was easier to use by the patient resulting in higher patient motivation and adaptation.

In a randomized controlled trial comparing the effectiveness of dental floss and interdental brushes in reducing signs of peri-implant mucositis, only patients with good manual dexterity were able to obtain inflammation reduction of the peri-implant tissues when the dental floss was used. Here, patients reported greater difficulty in handling dental floss and required more time when compared to the interdental brush [25]. Other studies reported high patient preference of the interdental brush due to its ease of use and minor discomfort [38].

Poor restoration designs and over-contoured implant restorations have been strongly linked to the prevalence of per-implant diseases. Having an emergence angle of more than 30 degrees is represents an important risk factor for peri-implantitis, especially in bone-level implants [52]. In the current review, only one of the included studies investigated the interproximal emergence angle and its influence on plaque accumulation and its role in the onset of peri-implant mucositis [25]. No relationship between both (emergence angle and inflammation of peri-implant tissues) could be found [25]. The reason may be that all implant restorations investigated in the study had an emergence angle of 25 degrees or less, which confirms the information stated previously by Yi et al. [52]. Similarly, other prosthetic designs such as splinted crowns may restrict interdental access and create sites that are more difficult to maintain compared to single crowns. However, the majority of included studies in this review involved implants restored with single crowns, and no comparative data were available on other prosthetic designs. This represents a limitation of the current evidence base, underscoring the need for future trials to systematically report and evaluate the role of restoration contour and design in relation to hygiene outcomes.

Patient compliance with the recommended self-administered oral hygiene measures is one of the important factors for the long-term prevention of biological complications around implants [53]. Therefore, reinstructing and remotivating the patient, as well as guiding the patient with the most appropriate interproximal cleaning device a crucial factor for patient compliance. Previously mentioned factors such as manual dexterity, restoration design and accessibility should be individually considered when a hygiene protocol is recommended for the patient.

The primary limitations of this systematic review stem from the limited available evidence, which restricted the number of studies eligible for each type of interproximal hygiene measure, with some measures being represented by only a single study. Additionally, substantial heterogeneity was observed across studies, likely due in part to variations in outcome measurements. The absence of a standardized definition of peri-implant mucositis in several studies may have further affected the reported results. Most clinical effects were assessed over short follow-up periods (14 days to 3 months), with only a few studies extending observations to medium-term follow-ups (6–12 months). A major limitation was the frequent absence of control groups, which considerably hindered the feasibility of conducting a meta-analysis. Furthermore, most of the included trials evaluated implants restored with single crowns, and no data were available on other prosthetic designs. With regard to jaw location, only two studies specified implant placement in the posterior region (one in the maxilla and one in the mandible), but neither provided stratified outcomes.

The current literature clearly highlights a substantial gap in evidence concerning self-administered oral hygiene practices around dental implants, especially in terms of interproximal devices. To date, recommendations for self-performed home care around dental implants have largely been based on evidence from periodontal studies conducted on natural teeth. However, implant-supported restorations frequently feature different anatomical contours and prosthetic designs, which can affect access for hygiene. Additionally, unlike natural teeth, implants are not susceptible to interproximal caries—an important consideration which raises doubts about whether cleaning methods like flossing, developed for natural teeth, are really suitable for dental implants such as flossing, from tooth-based to implant-based care. Given these prosthetic and anatomical differences, as well as the absence of caries risk, it is essential to reevaluate the relevance and effectiveness of traditional interproximal aids like dental floss in implant maintenance. Therefore, a consensus on the optimal patient-performed oral hygiene protocol for implant-supported restorations is required.

Future randomized controlled trials evaluating patient-administered interproximal hygiene interventions for managing peri-implant mucositis should incorporate larger sample sizes and a minimum follow-up of six months. Studies must include a control group using toothbrushing alone, and clearly define clinical criteria for diagnosing peri-implant mucositis in accordance with the most recent classification of peri-implant diseases.

## Conclusion

Based on the limited evidence available, interproximal cleaning devices demonstrated the potential to enhance mechanical biofilm control and reduce peri-implant mucosal inflammation when used in addition to toothbrushing. While some studies suggested a trend toward greater reduction in signs of peri-implant mucositis with oral irrigators and interdental brushes compared to dental floss, the evidence is insufficient to draw firm conclusions or establish superiority among devices. Dental professionals should continue to emphasize the importance of patient-administered interproximal hygiene as a critical component of long-term implant maintenance. The choice of device should be individualized, taking into account factors such as patient dexterity, prosthetic design, and accessibility. Further rigorously designed randomized clinical trials are needed to clarify the comparative effectiveness of different interproximal hygiene aids on peri-implant health.

## Data Availability

The datasets used and/or analysed during the current study are available from the corresponding author on reasonable request.

## Abbreviations

BOP: Bleeding on probing
CHX: Chlorhexidine
DF: Dental floss
GI: Gingival index
IDB: Interdental brush
IL: Interleukin
PI: Plaque index
TB: Toothbrush
WF: Water flosser
